# Analysis of Infectious Keratitis Isolates and Antimicrobial Resistance: An 8-Year Retrospective Study in Southern China

**DOI:** 10.3390/antibiotics15060615

**Published:** 2026-06-17

**Authors:** Jiayi Zheng, Jingyu Liao, Xinlei Zhao, Huijing Huang, Kaili Wu, Fang Duan

**Affiliations:** 1 State Key Laboratory of Ophthalmology, Zhongshan Ophthalmic Center, Sun Yat-sen University, Guangzhou 510060, China; 2 Guangdong Provincial Key Laboratory of Ophthalmology and Visual Science, Zhongshan Ophthalmic Center, Sun Yat-sen University, Guangzhou 510060, China; 3 Guangdong Provincial Clinical Research Centre for Ocular Diseases, Zhongshan Ophthalmic Center, Sun Yat-sen University, Guangzhou 510060, China; 4 State Key Laboratory of Ophthalmology, Optometry and Visual Science, Eye Hospital, Wenzhou Medical University, Wenzhou 325027, China

**Keywords:** infectious keratitis, microbial profiles, antimicrobial resistance

## Abstract

Objectives: To characterise the bacterial and fungal spectrum of infectious keratitis (IK) in southern China and to evaluate changes in the bacterial profiles and antimicrobial resistance (AMR) over an 8-year period (2017–2024). Methods: This retrospective study included patients with culture-positive IK treated between 2017 and 2024. Corneal scrapings were obtained for microbiological culture and pathogen identification. Antimicrobial susceptibility testing was performed for all bacterial isolates. Microbial distribution and in vitro antibiotic susceptibility were analysed. Results: A total of 2785 microbial isolates were recovered from 2741 patients. Overall, fungal isolates predominated (59.6%), exhibiting a distinct seasonal distribution, with *Fusarium* (40.2%) and *Aspergillus* (14.3%) being the most common genera. Among bacterial isolates, Gram-positive organisms were predominant (63.6%). The most frequently identified Gram-positive organisms were coagulase-negative staphylococci (CNS; 34.9%), while *Pseudomonas* (18.9%) was the most common Gram-negative pathogen. Over the study period, an increase in the proportions of CNS (*p* < 0.001) and *Serratia* was observed (*p* = 0.017), alongside a decline in *Pseudomonas* (*p* = 0.009) and *Kocuria* (*p* < 0.001). Resistance among Gram-positive isolates increased for penicillin (from 62.3% to 74.4%; *p* = 0.002) and levofloxacin (from 26.4% to 46.9%; *p* < 0.001), whereas Gram-negative resistance generally declined. Conclusions: IK in southern China is characterised by persistent fungal predominance and evolving bacterial composition. AMR patterns differ between Gram-positive and Gram-negative organisms, reflecting both shifts in pathogen distribution and species-specific resistance changes, highlighting the importance of continued regional surveillance to guide empirical therapy.

## 1. Introduction

Infectious keratitis (IK) is a sight-threatening corneal infection that develops following disruption of the corneal epithelial barrier and subsequent microbial invasion. Microbial proliferation together with the ensuing host inflammatory response can result in corneal destruction, visual impairment, and irreversible blindness. The incidence of IK is greater in developing countries than in developed countries [1]; it varies from 11.0 to 27.6 cases per 100,000 in the United States and 3.6 to 40.3 per 100,000 in the UK [2], with an estimated rate of 0.66 per 10,000 in Queensland, Australia [3]; in contrast, it rises dramatically to 113–799 in developing regions such as India and Nepal [2]. The causative organisms include bacteria, fungi, parasites, and viruses. Although bacterial keratitis accounts for the majority of cases, fungal keratitis remains a major contributor in many endemic settings [1]. The microbial aetiology varies across regions and is shaped by climatic, environmental, and socioeconomic factors [2]. Bacterial keratitis is predominant in certain regions of Europe and North America [4,5], whereas fungal keratitis is encountered more frequently in several regions of Asia and Africa [2,6].

Prompt and appropriate management is critical to preserving vision in patients with IK. Empirical treatment is typically initiated with broad-spectrum antimicrobial agents and subsequently adjusted according to microbiological findings, antimicrobial susceptibility profiles, and clinical response [1]. However, the widespread use of antibiotics has accelerated the emergence of antimicrobial resistance (AMR), which is recognized as a major public health threat that adversely affects treatment outcomes and imposes substantial social and economic burdens [7]. Excessive and inappropriate antibiotic usage is recognized as a major driver of AMR. Antibiotic consumption increased during the COVID-19 pandemic, with up to 72% of hospitalised patients receiving antibiotic treatment [8]. Although data regarding ophthalmic antibiotic use remain scarce, studies from Asia have documented increasing trends of ocular AMR during the pandemic period [9,10]. These emerging challenges reinforce the importance of the Global Action Plan on AMR adopted by the World Health Assembly in 2015 [11], which advocates continuous regional surveillance to support antimicrobial stewardship and inform empirical treatment strategies. Establishing an up-to-date understanding of regional AMR trends is essential for guiding treatment decisions and supporting future antimicrobial stewardship efforts.

This study retrospectively analysed the demographic, microbiological, and AMR data from 2741 cases of IK (2785 isolates) collected between 2017 and 2024. Comparisons between the 2017–2020 and 2021–2024 periods were performed to characterise temporal shifts in bacterial epidemiology and resistance patterns, thereby providing updated evidence to guide the clinical management of IK.

## 2. Results

Among the 2741 culture-positive cases in this study, 1169 cases were diagnosed during 2017–2020 and 1572 during 2021–2024. The demographic characteristics of the study population are summarised in Table 1. The median age was higher in 2021–2024 than in 2017–2020 (*p* < 0.001). Male patients accounted for the majority of cases, and no difference in sex distribution was observed between the two periods.

### 2.1. Isolated Microorganisms

**Figure 1:**
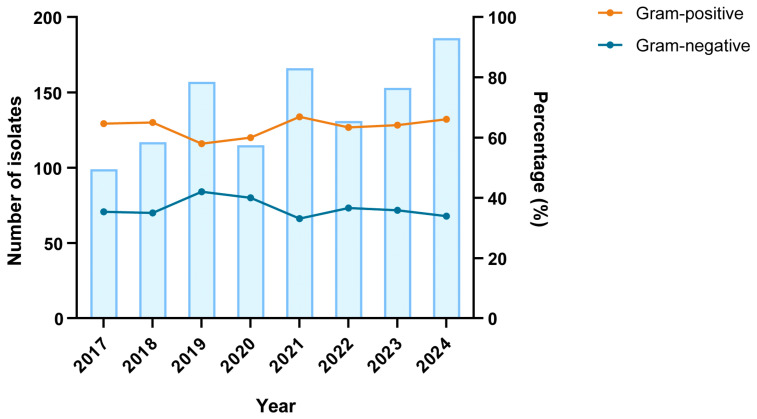
Annual number of bacterial isolates and the proportion of Gram-positive and Gram-negative bacteria from 2017 to 2024. The left Y-axis represents the number of isolates (displayed by bars). The right Y-axis represents the percentage of Gram-positive or Gram-negative bacteria (displayed by points and lines). They share the same X-axis, representing years.

A total of 2785 isolates were identified, including 1124 bacterial isolates (40.4%) and 1661 fungal isolates (59.6%). Gram-positive bacteria accounted for the majority of bacterial isolates throughout the study period (Figure 1). Although the proportion fluctuated annually, declining to 58.0% in 2019, it increased slightly from 64.6% in 2017 to 66.1% in 2024. Conversely, the proportion of Gram-negative bacteria decreased from 35.4% to 33.9% over the same period. Among Gram-positive isolates, *Staphylococcus epidermidis* was identified most frequently (23.5%, 264/1124), and *Pseudomonas aeruginosa* represented the most common Gram-negative species (17.6%, 198/1124).

There were 488 (43.4%) bacterial isolates identified during 2017–2020, while 636 (56.6%) were identified during 2021–2024. Comparison of bacterial profiles between the two periods showed higher proportions of coagulase-negative staphylococci (CNS) and *Serratia* isolates in 2021–2024, whereas lower proportions of *Pseudomonas* and *Kocuria* were observed. Detailed results are presented in Table 2.

**Figure 2:**
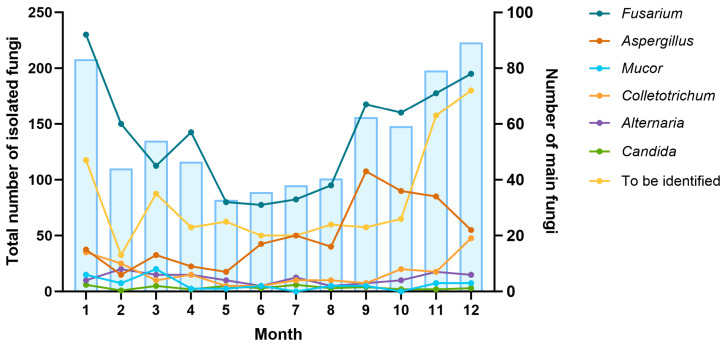
Monthly trends of cultured fungi associated with infectious keratitis from January 2017 to December 2024. The left Y-axis represents the total number of fungal isolates, displayed by bars. The right Y-axis represents the number of main fungal genus, displayed by lines. They share the same X-axis, representing months.

Table 3 presents the distribution of fungal isolates. *Fusarium* species were the most frequently isolated fungi (40.2%), followed by *Aspergillus* (14.3%) and *Colletotrichum* (5.0%). A seasonal distribution of fungal isolates was observed (Figure 2). The number of fungal isolates increased from September to December and remained comparatively low between May and July. This pattern largely reflected the distribution of *Fusarium* isolates, which constituted the most frequently recovered genus.

Polymicrobial infection, defined as the isolation of at least two different microorganisms, was identified in 44 cases (1.6%). All cases involved two pathogens. Mixed fungal–bacterial infection represented the most common pattern (50.0%). Polybacterial infections were observed in 19 patients (43.2%), while polyfungal infections occurred in only 3 patients (6.8%). CNS (25/44, 56.8%) were the most frequently identified bacteria in polymicrobial cases, while *Fusarium* and *Aspergillus* were the most frequently identified fungal genera (both 8/44, 18.2%). The proportion of polymicrobial infections increased from 0.9% in 2017–2020 to 2.2% in 2021–2024 (*p* = 0.007). Detailed information is provided in Supplementary Table S1.

### 2.2. Antimicrobial Resistance of Bacteria

Table 4 summarises the AMR profiles of Gram-positive and Gram-negative bacterial isolates between 2017–2020 and 2021–2024. Among Gram-positive isolates, resistance to penicillin and levofloxacin increased from 62.3% to 74.4% (*p* = 0.002) and from 26.6% to 46.9% (*p* < 0.001), respectively. Among Gram-negative isolates, resistance to several agents decreased, including piperacillin, cefuroxime, ceftazidime and amikacin. AMR patterns of the predominant bacterial taxa were analysed separately (Supplementary Tables S2 and S3). An increase in levofloxacin resistance was observed among CNS, while the resistance rate to ceftazidime in *Pseudomonas* decreased. Interpretation of AMR trends in *Streptococcus* and *Staphylococcus aureus* was limited by the small number of isolates tested.

## 3. Discussion

The present study examined microbial profiles and AMR patterns among infectious keratitis isolates collected over an eight-year period in southern China. A predominance of fungal pathogens was observed, together with temporal shifts in bacterial composition and AMR patterns.

Fungal isolates accounted for the majority of cultured pathogens, consistent with reports from other developing regions with substantial agricultural populations, such as India [12]. This distribution differs from that reported in developed countries, including the United States and European countries, where bacterial keratitis is the predominant aetiology [13,14,15]. The observed distribution may reflect the subtropical climate and agricultural setting of the study region [2,16]. By comparison, temperate regions such as North China and Romania report a markedly lower proportion of fungal isolates (7.01–8.87%) [17,18]. Regarding the geographic distribution of fungal genera, *Fusarium* was the most frequently isolated fungal genus (40.2%) in our study, similar to observations from central China and southern India [19,20], whereas *Candida* have been reported more frequently in temperate regions such as Canada and Spain [21,22]. In addition, a seasonal pattern of fungal keratitis was observed, largely attributable to *Fusarium*, with higher isolation rates from September to January and lower rates from May to July, consistent with reports from South Asia [2], which may be related to seasonal increases in airborne fungal concentrations during harvest periods [23]. These findings underscore the high risk of fungal keratitis in populations with frequent agricultural exposure and, clinically, highlight the need for heightened vigilance for fungal keratitis among clinicians in southern China and early initiation of antifungal therapy when clinically indicated.

In this study, Gram-positive bacteria were predominant (63.6%), with CNS and *Pseudomonas* being the most frequently recovered Gram-positive and Gram-negative bacteria, respectively, consistent with previous reports [4,5,24,25]. Temporal changes in bacterial composition were observed, including increased proportions of CNS and *Serratia* and reduced proportions of *Pseudomonas* and *Kocuria*. A rise in CNS has also been reported in Southern California [26], while a higher proportion of *Serratia* has been described in studies conducted during the COVID-19 period [14,27]. Although these observations may indicate temporal changes associated with the pandemic period, the underlying causes are likely multifactorial and require further investigation. In contrast, *Pseudomonas* exhibited a declining trend, in agreement with earlier reports [28,29]. Changes in contact lens practices and hygiene behaviours may have contributed to this pattern. Given that *Pseudomonas* keratitis is predominantly linked to contact lens wear, evidence suggests that the shift towards daily disposable lenses contributes to a reduced risk of *Pseudomonas* infection [30]. Additionally, pandemic-related lifestyle changes may have contributed to a transient reduction in contact lens usage [31]. A lower proportion of *Kocuria* was also observed; however, comparable longitudinal data remain limited. Together, these observations suggest a dynamic and continually evolving microbial profile of IK, underscoring the importance of constant regional surveillance.

The proportion of polymicrobial infections increased from 0.9% in 2017–2020 to 2.2% in 2021–2024. Polymicrobial keratitis has been associated with greater therapeutic complexity and less favourable visual outcomes, and advanced age and corticosteroid exposure have previously been identified as risk factors [32,33]. These findings support careful microbiological investigation and prudent corticosteroid use in patients at increased risk of polymicrobial infection.

Resistance among Gram-positive isolates increased during the study period, whereas resistance among Gram-negative isolates generally declined. The increase in penicillin resistance was consistent with reports from the United Kingdom [13], and a similar rise in levofloxacin resistance has been documented in Germany [34]. The extensive use of levofloxacin in ophthalmic practice may have contributed to this pattern. However, interpretation of resistance trends at the Gram-positive and Gram-negative group level requires caution, as temporal changes in pathogen composition can influence aggregate resistance estimates. To distinguish compositional effects from species-specific resistance changes, analyses were performed for the predominant isolates. CNS exhibited a significant increase in levofloxacin resistance during the later study period, whereas ceftazidime resistance in *Pseudomonas* decreased significantly over time. These findings indicate that the AMR shifts observed at the population level were shaped not only by changes in the relative prevalence of individual pathogens but also by evolving AMR patterns among specific species. Nevertheless, interpretation of species-specific trends remains constrained by the limited number of isolates available for some organisms, and continued surveillance will be required to verify these patterns. Different trends in resistance between Gram-positive and Gram-negative groups may be related to both distinct mechanisms of fluoroquinolone resistance acquisition and differing patterns of antibiotic exposure on the ocular surface, where Gram-positive organisms predominate and therefore may experience different antimicrobial selection pressures [35,36]. Among Gram-negative isolates, resistance remained low for several commonly used agents and declined over time for ceftazidime. This finding contrasts with the increasing cephalosporin resistance reported in Toronto and eastern England [14,37]. The discrepancy between regions further emphasises the value of local surveillance data when selecting empirical therapy. In our cohort, Gram-negative isolates retained high in vitro susceptibility to ceftazidime, aminoglycosides, carbapenems, and fluoroquinolones, supporting the continued use of these agents in the empirical treatment of Gram-negative keratitis.

A low resistance rate was observed for gatifloxacin, a fourth-generation fluoroquinolone. The overall resistance rate was 6.2%, with rates of 13.5% among Gram-positive isolates and 1.7% among Gram-negative isolates. These values were lower than those observed for levofloxacin. Although the number of isolates tested against gatifloxacin is limited, resistance among Gram-positive isolates was also lower than that observed for moxifloxacin (20.4%). A similar susceptibility profile has been reported in Shandong, China [24], suggesting that gatifloxacin may remain a useful option for initial empirical therapy against bacterial keratitis, although further surveillance is warranted.

This study characterised the microbial spectrum and antimicrobial resistance patterns of infectious keratitis over an eight-year period at a major ophthalmic centre in southern China. Several limitations should be acknowledged. First, the retrospective single-centre design may limit the generalisability of the findings to other regions of southern China. Second, nearly a quarter of the fungal isolates could not be identified to the genus level due to the lack of routine molecular sequencing for strains that exhibited atypical morphology or were absent from the VITEK MS database. Additionally, the number of isolates tested for susceptibility to certain antibiotics, particularly gatifloxacin, was limited, and antifungal susceptibility data were excluded due to the limited number of agents routinely tested. These limitations should be considered when interpreting the findings. Further multicentre studies with broader microbiological and susceptibility testing are required to validate the observed trends.

## 4. Materials and Methods

The study adhered to the principles of the Helsinki Declaration and was approved by the Institutional Ethics Committee of the Zhongshan Ophthalmic Center, Sun Yat-sen University. Due to the retrospective design, the requirement for patient consent was waived.

### 4.1. Data Collection

Electronic medical records were searched for the period from January 2017 to December 2024 using search terms including ‘corneal infection’, ‘ulcer’, ‘keratitis’, ‘bacterial keratitis’, ‘fungal keratitis’, and ‘corneal perforation’. From the identified records, the study included 2741 patients with culture-proven bacterial or fungal keratitis. Demographics and microbiological data were extracted.

### 4.2. Sample Collection and Processing

Corneal scrapings were obtained from all patients with a clinical diagnosis of IK at presentation. Specimens were immediately inoculated onto blood agar, chocolate agar, and potato dextrose agar, and subjected to Gram staining, KOH wet mount, and fluorescent staining. Following specimen collection, empirical antimicrobial therapy was initiated, typically comprising broad-spectrum topical antimicrobial agents selected according to the clinical judgment and smear findings. Treatment regimens were subsequently adjusted according to culture results, antimicrobial susceptibility testing, and clinical response during follow-up.

### 4.3. Microbiological Identification and Susceptibility Testing

Bacterial colonies were identified using the VITEK 2 Compact system (BioMérieux, Marcy-l’Étoile, France). Fungal isolates were identified based on morphological characteristics via microscopy and supplemented by the VITEK MS system when applicable. The clinical significance of isolated organisms was then determined through an integrated assessment of clinical presentation, direct smear findings, and microbiological characteristics, in accordance with the *Practical Guidance for Clinical Microbiology Laboratories: Diagnosis of Ocular Infections* [38]. Antimicrobial susceptibility testing was primarily performed using the VITEK 2 system (automated broth microdilution). For organisms not covered by the VITEK panels, supplementary testing was conducted using the Kirby–Bauer disk diffusion method or manual broth microdilution. Results were interpreted as susceptible, intermediate, or resistant. Results were interpreted according to the CLSI M100 edition in use at the time the isolate was tested (27th–34th editions). Due to practical constraints, not all antimicrobial agents were tested for every isolate.

### 4.4. Statistical Analysis

Data analysis was conducted using SPSS (version 25.0; IBM, Armonk, NY, USA). Continuous variables were expressed as medians (interquartile ranges) and compared using the Mann–Whitney U test, while categorical variables were presented as percentages and analysed by the Chi-square test or Fisher’s exact test. A *p* value of <0.05 was considered statistically significant.

## 5. Conclusions

In conclusion, this long-term study demonstrated a predominance of fungal pathogens and documented temporal changes in bacterial composition and AMR patterns among infectious keratitis isolates in southern China. Changes in overall resistance profiles appeared to reflect both shifts in bacterial composition and evolving resistance patterns within individual bacterial species. An increase in polymicrobial infections was also observed, potentially adding complexity to clinical management. Gatifloxacin resistance remained low throughout the study period, suggesting that it remains a reasonable option for empirical treatment. Ongoing surveillance of microbial epidemiology and antimicrobial susceptibility remains necessary to inform empirical therapy and antimicrobial stewardship in infectious keratitis.

## Figures and Tables

**Table 1:** Demographics of patients diagnosed with bacterial or fungal keratitis during 2017–2020 and 2021–2024.

		Overall (*n* = 2741)	2017–2020 (*n* = 1169)	2021–2024 (*n* = 1572)	*p* Value
Age (years)		56 (47–64)	54 (45–63)	57 (48–65)	<0.001 ^#^
Sex	Male (%)	1729 (63.1)	732 (62.6)	997 (63.4)	0.666 *
	Female (%)	1012 (36.9)	437 (37.4)	575 (36.6)	

^#^: Mann–Whitney U test. *: Chi-square test.

**Table 2:** Comparison of bacterial isolates in patients with infectious keratitis between 2017–2020 and 2021–2024.

Organisms	Total *n* (%), *n* = 1124	2017–2020 *n* (%), *n* = 488	2021–2024 *n* (%), *n* = 636	*p* Value *
Gram-positive	715 (63.6)	300 (61.5)	415 (65.3)	0.192
*Staphylococcus*	462 (41.1)	165 (33.8)	297 (46.7)	<0.001
coagulase-negative staphylococci	392 (34.9)	140 (28.7)	252 (39.6)	<0.001
*Staphylococcus aureus*	70 (6.2)	25 (5.1)	45 (7.1)	0.179
*Streptococcus*	88 (7.8)	45 (9.2)	43 (6.8)	0.128
*Corynebacterium*	34 (3.0)	16 (3.3)	18 (2.8)	0.663
*Micrococcus*	14 (1.2)	5 (1.0)	9 (1.4)	0.558
*Bacillus*	24 (2.1)	11 (2.3)	13 (2.0)	0.809
*Kocuria*	10 (0.9)	10 (2.0)	0 (0.0)	<0.001 ^#^
*Propionibacterium*	23 (2.0)	12 (2.5)	11 (1.7)	0.392
*Enterococcus*	11 (1.0)	6 (1.2)	5 (0.8)	0.546 ^#^
Others	49 (4.4)	30 (6.1)	19 (3.0)	
Gram-negative	409 (36.4)	188 (38.5)	221 (34.7)	0.192
*Pseudomonas*	212 (18.9)	109 (22.3)	103 (16.2)	0.009
*Acinetobacter*	23 (2.0)	12 (2.5)	11 (1.7)	0.392
*Serratia*	28 (2.5)	6 (1.2)	22 (3.5)	0.017
*Stenotrophomonas*	12 (1.1)	5 (1.0)	7 (1.1)	0.902
*Achromobacter*	10 (0.9)	5 (1.0)	5 (0.8)	0.754 ^#^
*Klebsiella*	13 (1.2)	3 (0.6)	10 (1.6)	0.137
*Moraxella*	11 (1.0)	3 (0.6)	8 (1.3)	0.366 ^#^
Others	100 (8.9)	45 (9.2)	55 (8.6)	

*: Chi-square test. ^#^: *p*-value was calculated using Fisher’s exact test.

**Table 3:** Genus distribution of fungal isolates of infectious keratitis from 2017 to 2024 at Zhongshan Ophthalmic Center.

Fungus	Count (%)
*Fusarium*	668 (40.2)
*Aspergillus*	238 (14.3)
*Colletotrichum*	83 (5.0)
*Alternaria*	57 (3.4)
*Candida*	42 (2.5)
*Mucor*	31 (1.9)
*Penicillium*	24 (1.4)
*Purpureocillium*	24 (1.4)
*Scedosporium*	18 (1.1)
*Acremonium*	14 (0.8)
*Curvularia*	13 (0.8)
*Botryosphaeria*	9 (0.5)
*Cladosporum*	8 (0.5)
Others	41 (2.5)
To be identified	391 (23.5)
Total	1661 (100.0)

**Table 4:** Summary of antimicrobial resistance profiles of bacterial isolates from infectious keratitis (2017–2020 vs. 2021–2024).

Organisms	2017–2024 *n*/*N* (%)	2017–2020 *n*/*N* (%)	2021–2024 *n*/*N* (%)	*p* Value *
Gram-positive				
Penicillin	428/614 (69.7)	149/239 (62.3)	279/375 (74.4)	0.002
Benzylpenicillin	267/452 (59.1)	97/157 (61.8)	170/295 (57.6)	0.392
Cefuroxime	13/115 (11.3)	13/115 (11.3)	0/0 (-)	-
Erythromycin	352/552 (63.8)	106/174 (60.9)	246/378 (65.1)	0.345
Clindamycin	178/456 (39.0)	46/123 (37.4)	132/333 (39.6)	0.663
Gentamicin	44/483 (9.1)	14/162 (8.6)	30/321 (9.3)	0.800
Tobramycin	126/194 (64.9)	124/192 (64.6)	2/2 (100.0)	0.542 ^#^
Amikacin	69/118 (58.5)	69/116 (59.5)	0/2 (0.0)	0.170 ^#^
Ciprofloxacin	210/509 (41.3)	69/169 (40.8)	141/340 (41.5)	0.890
Levofloxacin	245/647 (37.9)	77/289 (26.6)	168/358 (46.9)	<0.001
Moxifloxacin	100/490 (20.4)	30/169 (17.8)	70/321 (21.8)	0.290
Gatifloxacin	5/37 (13.5)	0/1 (0.0)	5/36 (13.9)	1.000 ^#^
Rifampicin	40/459 (8.7)	12/163 (7.4)	28/296 (9.5)	0.446
Vancomycin	2/569 (0.4)	0/173 (0.0)	2/396 (0.5)	1.000 ^#^
Linezolid	4/480 (0.8)	3/160 (1.9)	1/320 (0.3)	0.110 ^#^
Gram-negative				
Piperacillin	48/324 (14.8)	29/150 (19.3)	19/174 (10.9)	0.034
Cefuroxime	111/162 (68.5)	88/115 (76.5)	23/47 (48.9)	<0.001
Ceftazidime	63/356 (17.7)	35/151 (23.2)	28/205 (13.7)	0.020
Imipenem	36/335 (10.7)	21/152 (13.8)	15/183 (8.2)	0.098
Meropenem	20/343 (5.8)	11/150 (7.3)	9/193 (4.7)	0.295
Gentamicin	49/346 (14.2)	23/152 (15.1)	26/194 (13.4)	0.647
Tobramycin	46/371 (12.4)	28/181 (15.5)	18/190 (9.5)	0.080
Amikacin	41/375 (10.9)	26/181 (14.4)	15/194 (7.7)	0.040
Ciprofloxacin	45/356 (12.6)	17/153 (11.1)	28/203 (13.8)	0.451
Levofloxacin	31/392 (7.9)	17/184 (9.2)	14/208 (6.7)	0.358
Gatifloxacin	1/60 (1.7)	0/0 (-)	1/60 (1.7)	-

*: Chi-square test. ^#^: *p*-value was calculated using Fisher’s exact test.

## Data Availability

The datasets used and analysed during the current study are available from the corresponding author on reasonable request.

## Supplementary Materials

The following supporting information can be downloaded at: https://www.mdpi.com/article/10.3390/antibiotics15060615/s1, Table S1: Microbial spectrum of polymicrobial keratitis isolates at Zhongshan Ophthalmic Center (2017–2024). Table S2: Comparisons of the antimicrobial resistance of the most prevalent Gram-positive bacteria isolated from infectious keratitis between 2017–2020 and 2021–2024. Table S3: Comparison of antimicrobial resistance profiles of *Pseudomonas* isolated from infectious keratitis between 2017–2020 and 2021–2024.
