# Supplementary material for: Analysis of Infectious Keratitis Isolates and Antimicrobial Resistance: An 8-Year Retrospective Study in Southern China
Source: Antibiotics (Basel). 2026 Jun 17;15(6):615. doi: 10.3390/antibiotics15060615 (PMC13295968; doi:10.3390/antibiotics15060615)
Supplement: Supplementary file 1 [file antibiotics-15-00615-s001.zip › antibiotics-4314364-supplementary.pdf]

Supplementary Material

Table S1. Microbial spectrum of polymicrobial keratitis isolates at Zhongshan Ophthalmic Center (2017–2024)

Infection Type	Overall (n = 2741)	2017–2020 (n = 1169)	2021–2024 (n = 1572)	P value*
Polymicrobial Infection, n (%)	44(1.6)	10(0.9)	34(2.2)	0.007
Bacterial + fungal	22(0.8)	2(0.2)	20(1.3)	-
Polybacterial	19(0.7)	8(0.7)	11(0.7)	-
Polyfungal	3(0.1)	0(0.0)	3(0.2)	-

*: Chi-square test

Table S2. Comparisons of the antimicrobial resistance of the most prevalent Gram-positive bacteria isolated from infectious keratitis between 2017–2020 and 2021–2024

Antibiotics	coagulase-negative Staphylococci			Streptococcus			Staphylococcus aureus		
	2017-2020 n/N (%)	2021-2024 n/N (%)	P value*	2017-2020 n/N (%)	2021-2024 n/N (%)	P value*	2017-2020 n/N (%)	2021-2024 n/N (%)	P value*
Penicillin	91/104(87.5)	224/250(89.6)	0.565	12/39(30.8)	0/19(0.0)	0.005#	16/20(80.0)	39/44(88.6)	0.443#
Benzylpenicillin	88/133(66.2)	163/251(64.9)	0.810			-	9/24(37.5)	7/44(15.9)	0.045
Erythromycin	89/138(64.5)	170/252(67.5)	0.553	4/5(80.0)	34/41(82.9)	1.000#	8/25(32.0)	18/44(40.9)	0.463
Clindamycin	39/102(38.2)	110/250(44.0)	0.321			-	6/20(30.0)	16/44(36.4)	0.619
Gentamicin	11/137(8.0)	24/252(9.5)	0.623			-	3/25(12.0)	4/45(8.9)	0.694#
Levofloxacin	49/140(35.0)	151/252(59.9)	<0.001	6/43(14.0)	5/41(12.2)	0.811	5/25(20.0)	10/45(22.2)	0.828
Ciprofloxacin	60/138(43.5)	119/252(47.2)	0.478			-	5/25(20.0)	10/45(22.2)	0.828
Moxifloxacin	26/137(19.0)	62/252(24.6)	0.205	0/5(0.0)	0/24(0.0)	-	4/25(16.0)	8/45(17.8)	1.000#
Rifampicin	11/138(8.0)	28/251(11.2)	0.317			-	1/25(4.0)	0/45(0.0)	0.357#
Vancomycin	0/137(0.0)	0/250(0.0)	-	0/6(0.0)	0/41(0.0)	-	0/24(0.0)	0/44(0.0)	-
Linezolid	1/126(0.8)	1/249(0.4)	1.000#	0/5(0.0)	0/23(0.0)	-	2/23(8.7)	0/43(0.0)	0.118#

*: Chi-square test

#: P-value was calculated using Fisher's exact test

Table S3. Comparison of antimicrobial resistance profiles of *Pseudomonas* isolated from infectious keratitis between 2017–2020 and 2021–2024

Antibiotics	2017-2020 n/N (%)	2021-2024 n/N (%)	P value*
Piperacillin	14/107(13.1)	6/103(5.8)	0.073
Ceftazidime	17/107(15.9)	6/103(5.8)	0.020
Imipenem	7/108(6.5)	4/103(3.9)	0.396
Meropenem	2/107(1.9)	2/103(1.9)	1.000#
Tobramycin	4/109(3.7)	2/103(1.9)	0.684#
Gentamicin	7/108(6.5)	3/103(2.9)	0.333#
Amikacin	0/109(0.0)	0/103(0.0)	-
Levofloxacin	5/108(4.6)	4/103(3.9)	1.000#
Ciprofloxacin	5/108(4.6)	5/103(4.9)	1.000#

*: Chi-square test

#: P-value was calculated using Fisher's exact test
